# Partial Dissociation of Truncated Peptides Influences the Structural Dynamics of the MHCI Binding Groove

**DOI:** 10.3389/fimmu.2017.00408

**Published:** 2017-04-18

**Authors:** Olivier Fisette, Sebastian Wingbermühle, Lars V. Schäfer

**Affiliations:** ^1^Center for Theoretical Chemistry, Faculty of Chemistry and Biochemistry, Ruhr-University, Bochum, Germany

**Keywords:** major histocompatibility complex class I (MHCI), peptide loading complex (PLC), antigen, peptide editing, protein dynamics, molecular dynamics (MD) simulations

## Abstract

Antigen processing on MHCI involves the exchange of low-affinity peptides by high-affinity, immunodominant ones. This peptide editing process is mediated by tapasin and ERAAP at the peptide C- and N-terminus, respectively. Since tapasin does not contact the peptide directly, a sensing mechanism involving conformational changes likely allows tapasin to distinguish antigen-loaded MHCI molecules from those occupied by weakly bound, non-specific peptides. To understand this mechanism at the atomic level, we performed molecular dynamics simulations of MHCI allele B*44:02 loaded with peptides truncated or modified at the C- or N-terminus. We show that the deletion of peptide anchor residues leads to reversible, partial dissociation of the peptide from MHCI on the microsecond timescale. Fluctuations in the MHCI α_2−1_ helix segment, bordering the binding groove and cradled by tapasin in the PLC, are influenced by the peptide C-terminus occupying the nearby F-pocket. Simulations of tapasin complexed with MHCI bound to a low-affinity peptide show that tapasin widens the MHCI binding groove near the peptide C-terminus and weakens the attractive forces between MHCI and the peptide. Our simulations thus provide a detailed, spatially resolved picture of MHCI plasticity, revealing how peptide loading status can affect key structural regions contacting tapasin.

## 1 Introduction

**Figure 1:**
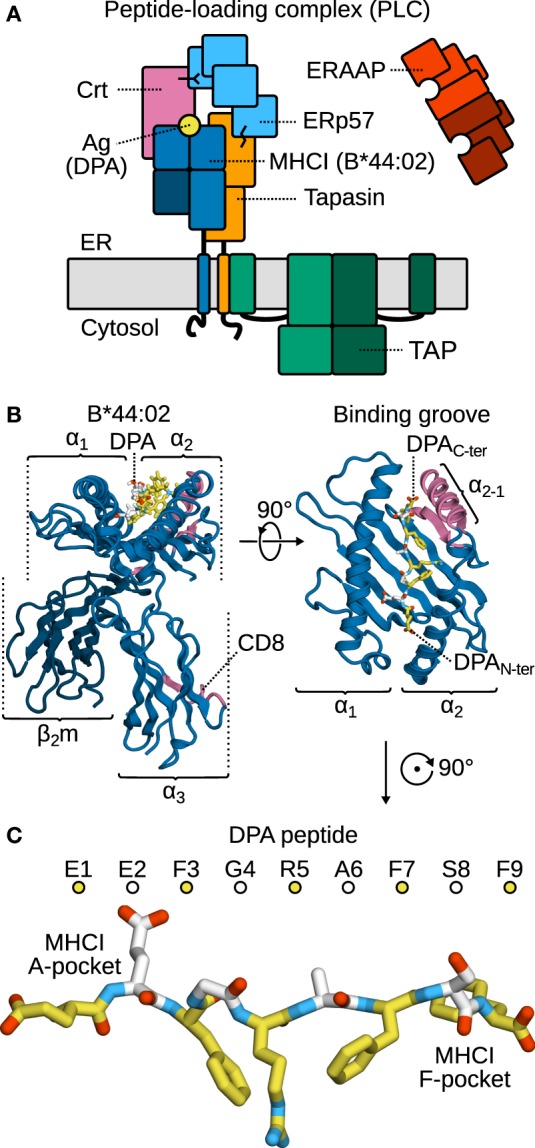
**The peptide-loading complex (PLC) and selected components**. **(A)** The PLC is assembled in the ER. Tapasin, a chaperone and peptide exchange catalyst, bridges the TAP transporter to MHCI. Accessory proteins ERp57 and calreticulin (Crt) stabilize the complex. ERAAP refines peptides through its peptidase activity. **(B)** MHCI consists of a heavy, variable α chain and the invariant, light β_2_m chain. The α_1_ and α_2_ domains form the peptide-binding groove, while α_3_ bears the CD8 recognition loop. Both the α_2−1_ helical segment and the CD8 loop contact tapasin in the PLC. **(C)** The antigen is bound to the MHCI groove in an extended conformation; the two groove regions interacting with the peptide N- and C-terminus are termed the A- and F-pockets, respectively. Alternating colors distinguish sequential residues.

To perform their signaling function at the cell surface, major histocompatibility complex class I (MHCI, Figure 1B) molecules (1–4) are first loaded with antigen peptides in the lumen of the endoplasmic reticulum (ER) by the peptide-loading complex (PLC, Figure 1A) (5), a large multi-protein assembly whose structural organization remains to be resolved at the atomic level. A central component of the PLC, tapasin (6, 7), edits the antigen repertoire exposed at the cell surface (8–10) by selecting peptides according to their C-terminus (11). To do so, tapasin accelerates the off-rate of MHCI-bound low-affinity cargo (12–14). Antigen candidates are thus rapidly exchanged until a high-affinity, immunodominant one binds (14, 15). Concomitantly, the aminopeptidase associated with antigen processing in the ER (ERAAP) (16–18) also edits the antigen repertoire, by cleaving peptides at their N-terminus (11) and thus adjusting their length so that they fit optimally into the MHCI binding groove (19).

According to the predicted structure of the tapasin–MHCI complex (14, 15), direct contact between tapasin and the MHCI-bound peptide is very unlikely. Indirect sensing and loading mechanisms have, therefore, been proposed to explain how tapasin influences peptide binding to MHCI. In a previous work (15), we have shown that the tapasin N-terminal domain (TN) acts on MHCI by pulling on its α_2−1_ region near the peptide C-terminus, thereby widening the binding groove and facilitating the release of low-affinity peptides. We showed that peptides compete with tapasin by pulling the same α_2−1_ region in the opposite direction, thus tightening the binding groove. Forces exerted by high-affinity peptides overcome those of tapasin, closing the groove and priming the PLC for dissociation (15). This is consistent with the observation that an engineered MHCI variant (K^b^Y84C) whose α_2−1_ region is linked to α_1_ by a disulfide bond, which can exert strong closing forces, is able to breach cellular quality control (20). However, other investigations have highlighted the importance of the MHCI α_3_ domain for peptide editing (21, 22), in line with α_3_ contacting tapasin at its C-terminal domain (TC). Allosteric coupling between α_3_- and the α_1_α_2_ binding groove domain has been suggested for chicken allele BF2*15:01 (23), and recently also been proposed for the human allele B*44:02 (24), prompting a mechanism according to which tapasin modulates binding groove plasticity through the TC–α_3_ interface. The direct and allosteric mechanisms are not mutually exclusive, though. They could be involved to different degrees, e.g., in different alleles, in the peptide editing function itself, or in the sensing of properly loaded MHCI.

Several molecular dynamics (MD) simulation studies have probed MHCI dynamics (25–35), how they correlate with peptide cargo (36–38), and their implications for peptide editing by tapasin (14, 15). These studies often compare peptide-loaded (MHCI^PL^) and -deficient (MHCI^PD^) molecules (14, 15, 25, 33), because crystal structures of MHCI complexed with low-affinity peptides are usually not available. MHCI^PD^ computational models can be easily prepared from crystal structures of high-affinity antigen-bound MHCI by peptide removal. This approach, however, might suffer from limitations. The PD state may not be particularly relevant since, *in vivo*, MHCI molecules are loaded with a peptide cargo before they are even recruited by tapasin (39). In the context of the PLC, tapasin stabilizes MHCI in a peptide-receptive state, hence accelerating peptide binding (9). Furthermore, peptide release is known to be the rate-limiting step in peptide editing (14). It follows that MHCI^PD^ is only a transient state, which rapidly leads to the binding of a new peptide. Hence, the essential role of tapasin is to distinguish between MHCI-bound peptides of different affinities, not between the PD and PL forms. MHCI^PD^ may, therefore, not be an optimal proxy for MHCI bound to low-affinity cargo. In that respect, a recent study involving truncated, low-affinity peptides is of particular interest (37). In that work, the authors used both MD simulations and experimental techniques to study the importance of termini residues in two peptides that bind to murine MHCI H-2K^b^. From 50 ns MD simulations, they reported increased binding groove RMSD and RMSF for the peptides truncated at either the N- or C-terminus. Thermal denaturation experiments carried out with the same truncated peptides yielded consistent results: the melting point (T_m_) of MHCI loaded with truncated peptides was significantly lowered. The peptide C-terminus was also shown to be involved in retaining MHCI at the cell surface.

In the present work, to better understand MHCI structure and dynamics in the context of peptide editing, we have performed microsecond-timescale MD simulations of MHCI allele B*44:02 bound to N- or C-truncated DPA (high-affinity antigen peptide derived from HLA-DPα; Figure 1C). This allows us to correlate MHCI plasticity, including local features of the binding groove, such as the structural stability of the α_2−1_ region, to specific changes in peptide cargo. We show that the peptide termini anchor it to the groove; removal of a single residue at the C-terminus suffices to trigger partial dissociation of the peptide, while removing two residues at the N-terminus has a similar effect. At the N-terminus, both the peptide main chain and the residue side chains affect stability. Conversely, the side chain of the terminal residue is critical for peptide stability at the C-terminus. Even in the full-length antigen, central residues are somewhat flexible and undergo thermal fluctuations, thus deviating from their position in the X-ray crystal structure over the course of the simulation. Any C-terminal deletion results in a widening of the binding groove near the F-pocket, similar to that observed in MHCI^PD^. Conversely, N-terminal deletions and peptide removal narrow the binding groove near the A-pocket. The overall structural stability of the groove is heavily influenced by the peptide cargo, with α_2−1_ being the most affected region, confirming the importance of tapasin TN as an MHCI chaperone. Long-range effects on MHCI plasticity could only be observed in one of the systems under study: B*44:02 loaded with an antigen truncated by a single residue at the N-terminus. In this specific case, the increased fluctuations in the α_1_α_2_ domain lead to increased fluctuations, and hence increased configurational entropy, in the α_3_ domain that contacts tapasin TC via the CD8 recognition loop. Using a C-truncated peptide as proxy for a low-affinity cargo, we performed additional simulations of MHCI in complex with tapasin. We show that tapasin modulates MHCI through an opening of the binding groove that reduces the attractive forces between MHCI and the peptide.

## 2 Materials and Methods

### 2.1 System Generation

Coordinates of B*44:02 loaded with the HLA DPA*0201 9-mer peptide were taken from a 1.6-Å resolution X-ray crystal structure (PDB ID 1M6O) (40). The α subunit contains 276 residues and excludes the membrane-spanning helix and cytosolic tail, while the invariant β_2_m subunit contains 99 residues. Water molecules resolved in the crystal structure were retained. PROPKA 3.1 (41) was used to determine protonation states at pH 7. D156 in the α_2−2_ helix segment was the only residue with a non-standard protonation state, sharing a proton with D114 in β_6_ at the bottom of the binding groove.

The structure was first subjected to 60 steps of steepest-descent (SD) energy minimization. Peptide removal, truncation, and side chain replacement were then used to generate from MHCI^PL^ the starting structures for MHCI^PD^, MHCI loaded with six truncated DPA peptides (Δ1N, Δ2N, Δ3N, Δ1C, Δ2C, Δ3C) and MHCI loaded with three peptides in which one or two residues were replaced by glycines (δ1N, δ2N, δ1C); all peptides were capped with standard (charged) amine and carboxyl termini. All systems were solvated in periodic rhombic dodecahedron cells, with a 10-Å minimal distance between the solute and the box edge. Random water molecules were replaced by Na^+^ and Cl^−^ ions at a 0.15 M concentration, yielding a neutral net charge. The systems contained ≈69,000 atoms in cells of ≈280,000 Å^3^. Finally, all systems were again subjected to 500 steps of SD energy minimization.

The tapasin–MHCI^Δ1C^ system was built from the results of a previous simulation of tapasin–MHCI (14, 15) in a solvated periodic rhombic dodecahedron cell with a 10-Å minimal distance between the solute and the box edge, and a 0.15 M concentration of Na^+^ and Cl^−^ ions. The system contained ≈140,000 atoms in a ≈1,480,000 Å^3^ cell. The DPA peptide in this system was truncated and capped with a carboxyl terminus prior to 500 steps of SD energy minimization.

### 2.2 MD Simulations

Simulations were carried out with GROMACS 2016.1 (42, 43). The Amber99SB*-ILDNP protein forcefield (44–49) and TIP3P water model (50) were used. The SETTLE (51) and LINCS (52) algorithms were applied to constrain the internal degrees of freedom of water molecules and the bonds in other molecules, respectively. In combination with virtual site hydrogens (53), this allowed for a 4-fs integration time step. Short-range non-bonded Coulomb and Lennard-Jones 6–12 interactions were treated with a Verlet-buffered pair list (54) with potentials smoothly shifted to zero at a 10-Å cutoff. Long-range Coulomb interactions were treated with the PME method (55) with a grid spacing of 1.2 Å and cubic spline interpolation. Analytical dispersion corrections were applied for energy and pressure to compensate for the truncation of the Lennard-Jones interactions. The thermodynamic ensemble was nPT. Temperature was kept constant at 300 K by a velocity-rescaling thermostat with a stochastic term (56), with coupling time constant 0.1 ps. For constant 1.0-bar pressure, an isotropic Berendsen barostat (57) was used with coupling time constant 0.5 ps and 4.5 × 10^−5^/bar compressibility.

For each system, five independent 1.0-µs trajectories were acquired (by generating random initial velocities from a Boltzmann distribution), with coordinates recorded every 10 ps. All of these simulations were preceded by 10 ns of equilibration, during which all heavy (non-hydrogen) protein atoms were position-restrained by harmonic potential energy functions with force constants of 1,000 kJ/(mol nm^2^).

### 2.3 Analysis

The first 10 ns of simulation without position restraints were considered further equilibration time and were not used for analysis of averaged RMSD, binding groove width, RMSF, configurational entropy, and pairwise forces between residues. In addition, one trajectory involving the Δ1C peptide was excluded from all analyses since the peptide dissociated entirely from the binding groove (see Results for further details).

Configurational entropies were calculated using the quasi-harmonic approximation (QHA) as formulated by Schlitter (58),

(1) $$S=0.5\;k_{\text{B}}\;ln\text{ det}\left(1+k_{\text{B}}\text{T}e^{2}\hbar^{-2}\;M^{1/2}\;C\;M^{1/2}\right)>S_{\text{true}},$$

which provides an upper bound to the configurational entropy. In this equation, *k_B_* is the Boltzmann constant, T the temperature, *e* Euler’s number, ℏ the reduced Planck constant, and **M** the 3N-dimensional diagonal mass matrix of the N particles. The matrix **C** is the covariance matrix of particle positions,

(2) $$C=\langle (x-\langle x\rangle ){\left(x-\langle x\rangle \right)}^{\text{T}}\rangle ,$$

where the 3N-dimensional vector **x** represents the Cartesian coordinates of the N particles for which the entropy is calculated after removing overall translation and rotation by fitting to a reference structure. The starting structure of our simulations was used as the reference structure for this fit. The coordinates of the C_α_ atoms were used to construct **C**. By comparing the entropy accumulated for the full 5.0 µs of sampling for each system to the value obtained when discarding a random 10% of the data, we estimated the error and verified that our sampling enables us to evaluate configurational entropies with sufficient statistical reliability.

Force distribution analysis (FDA) was performed using the GROMACS-PF2 implementation (59). Pairwise atom–atom forces were recomputed from the trajectories and were time- and residue-averaged. For the latter, the norm of the sum of the interatomic force vectors between all atoms i and j of residues v and w were calculated, ${\text{F}}_{\text{vw}}^{\text{res}}=|\sum_{\text{ij}}\;F_{ij}|$, with i ∈ v and j ∈ w. Attractive and repulsive forces were distinguished by sign.

## 3 Results

Starting from an X-ray crystal structure of MHCI allelle B*44:02 loaded with the specific, high-affinity HLA DPA*0201 peptide (40), we set up 8 simulation systems with different peptides. Our reference system is MHCI^PL^, containing the full-length 9-mer DPA peptide. To simulate lower-affinity peptides, we built 6 truncated systems; these contain the DPA peptide with one, two or three residues deleted at either the N- or the C-terminus, denoted by MHCI^Δ1N^ … MHCI^Δ3C^. An MHCI^PD^ system was also built by complete peptide removal. Trajectories totaling 5 µs of MD simulation (5 × 1 µs) were acquired for each system.

### 3.1 Partial Peptide Dissociation on the Microsecond Timescale

**Figure 2:**
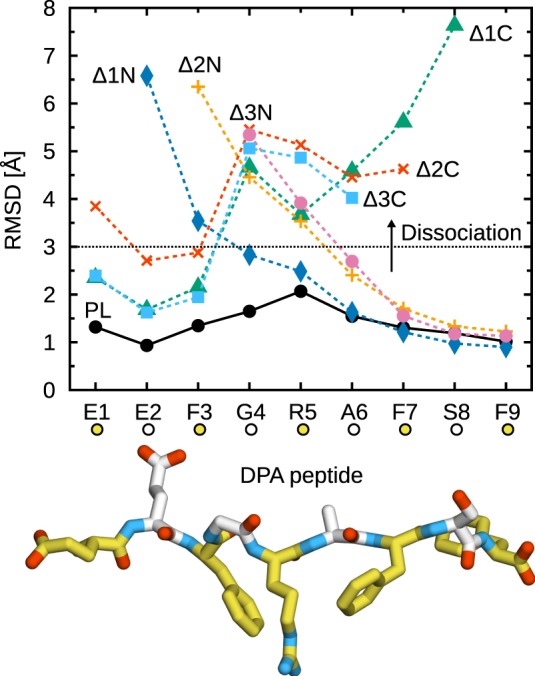
Partial dissociation of truncated peptides from B*44:02. C_α_ RMSD from the initial position in the X-ray crystal structure were averaged for each peptide residue over the five independent 1-µs trajectories acquired for each system. The dotted line represents a chosen dissociation threshold at 3 Å.

Figure 2 shows the C_α_ RMSD to the X-ray crystal structure for all studied peptides. Deletion of a single C-terminal residue (Δ1C) is sufficient to trigger partial peptide dissociation from the binding groove on the simulation timescale. The C-terminal F9 residue, whose side chain is buried in the F-pocket, is thus crucial for binding to B*44:02. In its absence, central residues cannot stabilize the peptide, and a large segment (G4–S8) dissociates from the MHCI binding groove (Figure 2, Δ1C). Further deletions (Δ2C, Δ3C) have no additional effect; the same G4–S8 region unbinds from the groove. These results are consistent with the observation (36) that removing or modifying the side chain of the peptide C-terminal residue accelerates dipeptide-catalyzed peptide exchange.

In contrast to the C-terminus, a single N-terminal deletion (Δ1N) has a less pronounced effect and leads only to the partial dissociation of residues E2–F3 (Figure 2). Removal of E2, which is buried in the A-pocket, is necessary for dissociation of the F3–R5 region, comparable to the loss of interactions observed for C-terminal deletions. Thus, the peptide is held in place by its termini at E2 and F9, while the residues in between contribute less to MHCI binding. The importance of peptide termini was shown previously from the thermal stability of MHCI molecules loaded with modified peptides (60). Our results are also consistent with the conclusions of a previous MD study of MHCI allele H2-K^b^ loaded with truncated peptides (37), although the shorter simulation times of that study precluded the observation of partial peptide dissociation.

Due to the thermal fluctuations, even the full-length DPA peptide deviates from its position in the X-ray crystal structure. While the N- and C-terminal residues remain firmly anchored to the A- and F-pocket, respectively, central residues G4, R5, and A6 exhibit an average C_α_ RMSD of about 1.5 Å, consistent with the idea that residues E2 and F9 are the main determinants of peptide–MHCI stability. In line with this observation, even though the dehydrated environment of the crystal might naturally favor a tight binding groove, crystallographic Debye–Waller factors (B-factors) are slightly higher for residues 3–8 than they are for E1, E2, and F9. This peptide flexibility, observed in our simulations of MHCI in solution, could have implications for antigen recognition. MHC recognition by TCR, reviewed recently (61), involves three TCR loops (CDR1, 2, and 3); while CDR1 and 2 contact MHC, CDR3 is located directly atop the antigen peptide and thus interacts mostly with residues at the center of the peptide. For instance, interaction hotspots at positions 4, 5, and 8 in a 9-mer peptide have been identified in the structure of a TCR complexed with an MHCI molecule loaded with a cytomegalovirus antigen (62). Conformational changes observed in CDR3 upon binding to MHCI^PL^ (61) were interpreted in terms of an induced fit mechanism that could be facilitated by small-scale rearrangements and fluctuations of the peptide central residues.

In all but one of the simulations performed in the present study, the peptide remained partially bound to B*44:02 and did not fully dissociate from the binding groove. However, in one of the Δ1C peptide simulations, the peptide C-terminus dissociated from the groove (after 5 ns), before the N-terminus also dissociated (after 255 ns). This trajectory was excluded from all further analyses. Investigating the possibility of complete peptide dissociation would require much more extensive simulations. To determine the thermodynamic equilibrium, one would need to fully sample both unbinding and rebinding events, which is clearly beyond the scope of the present work.

**Figure 3:**
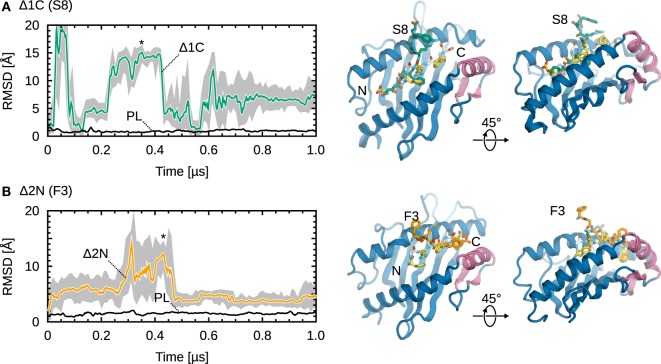
**Representative examples of partial dissociation events**. **(A)** C_α_ RMSD timeseries of MHCI-bound Δ1C S8 (from its initial position in the X-ray crystal structure). The MHCI^Δ1C^ structure shown is from a trajectory frame at t ≈ 0.35 µs; the corresponding point is labeled (*). **(B)** C_α_ RMSD timeseries of MHCI^Δ2N^ DPA F3. The structure shown is from a trajectory frame at t ≈ 0.43 µs. Black lines show the reference MHCI^PL^ system. 1-ns moving averages are overlaid on the timeseries (gray).

Partial dissociation is reversible, with truncated peptides mostly sampling conformations away from the binding groove, but also inside the groove to a lesser extent. Figure 3 shows RMSD timeseries from simulations of Δ1C and Δ2N, the two smallest deletions leading to partial dissociation of the central residues. For the C-terminally truncated peptide (Figure 3A), we observe three dissociations and two rebinding events; the peptide spends around 5% of the trajectory time with both termini bound. In the case of Δ2N (Figure 3B), the peptide spends roughly 35% of the trajectory time in the vicinity of the binding groove, although it never goes back to its initial position. Even though a similar behavior was observed in each of the five independent simulations, significantly longer trajectories would be required for statistically more accurate results. Larger peptide truncations (Δ2C, Δ3C, Δ3N) show a behavior similar to that of Δ2N, with the peptide sampling two sets of conformations, either away from or close to the groove.

Taken together, our results suggest that MHCI loaded with low-affinity peptides can exist in two states: one where both peptide termini are near or inside the binding groove, and another where one terminus is displaced from the groove (see structures in Figure 3). These results are consistent with NMR experiments of MHCI molecules loaded with variant peptides (63), which showed that MHCI at the cell surface exist as a mixture of a major state and a minor state, the former being more hydrated. Since the same study showed that the melting temperature of MHCI increases with the prevalence of the less hydrated, minor state, we speculate that partial peptide dissociation might be the first step in the internalization and recycling of surface-exposed MHCI molecules.

### 3.2 Influence of Peptide Truncation on B*44:02 Structure and Dynamics

Simulation studies of MHCI often compare dynamics in the PL and PD forms (14, 15, 25, 33). However, *in vivo*, MHCI molecules are bound to (usually low-affinity) peptides after their synthesis and assembly with β_2_m and calnexin (39), before peptide editing takes place. We compared the dynamics of the PD and PL forms to those of MHCI loaded with various truncated peptides to assess if MHCI^PD^ is an appropriate proxy for low-affinity peptides.

**Figure 4:**
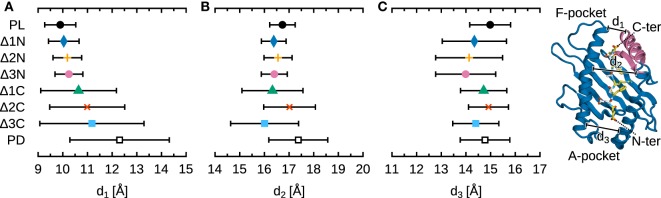
Influence of peptide cargo on B*44:02 binding groove width. Distances were averaged over all simulations for each system; standard deviation is shown as bars. Standard errors of the mean, obtained from a block averaging procedure (64), are below 0.5 Å in all cases. **(A)** Distance d_1_ is measured between the C_α_ atoms of Y85 and T138. **(B)** d_2_ between Y74 and V152. **(C)** d_3_ between Y59 and Y171.

Figure 4 shows the width of the B*44:02 binding groove, measured at three positions, as a function of peptide cargo. Peptide release widens the binding groove by 2.5 Å at d_1_, measured near the F-pocket, and by 0.5 Å at d_2_, measured at the center of the groove. However, no significant change is observed at d_3_, near the A-pocket. In addition to this opening, the amplitude of the fluctuations of the groove dimensions is larger in the absence of peptide. Interestingly, the effects of N-terminal deletions are very different from those of full peptide removal. The groove remains mostly unchanged at d_1_ (F-pocket) and d_2_ (center), but narrows by 1 Å at d_3_ (A-pocket). This partial collapse suggests that MHCI^PD^ is not an appropriate surrogate for studying the effects of N-terminal peptide editing. The effects of C-terminal deletions are more similar to those of peptide removal, albeit less pronounced. As more residues are removed, the binding groove widens at d_1_ (F-pocket), as expected, and fluctuations around the average increase at d_2_ (center); d_3_ (A-pocket) remains mostly unchanged. In conclusion, these results post a cautionary note when using MHCI^PD^ to study peptide editing; while it might be warranted for C-terminal editing by tapasin (since the binding groove widens in the PD form as it does for the ΔC peptides), groove dynamics near the N-terminus depend not only on the loading state of the A-pocket, but also on that of the opposite F-pocket.

**Figure 5:**
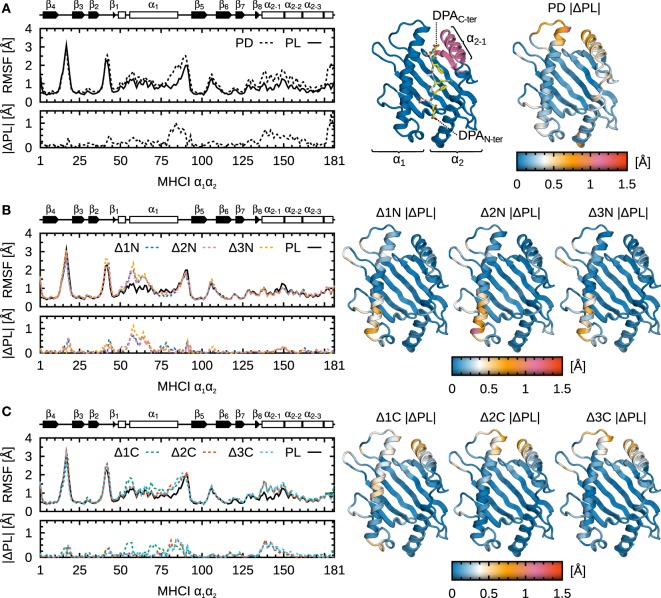
Influence of peptide cargo on B*44:02 α_1_α_2_ fluctuations. C_α_ RMSF along sequence and the absolute difference compared to MHCI^PL^ (|ΔPL|) are shown for each system. ΔRMSF are also mapped onto the structure of α_1_α_2_. Peptide not shown for clarity. **(A)** MHCI^PD^. **(B)** MHCI^Δ1N–Δ3N^. **(C)** MHCI^Δ1C–Δ3C^.

**Figure 6:**
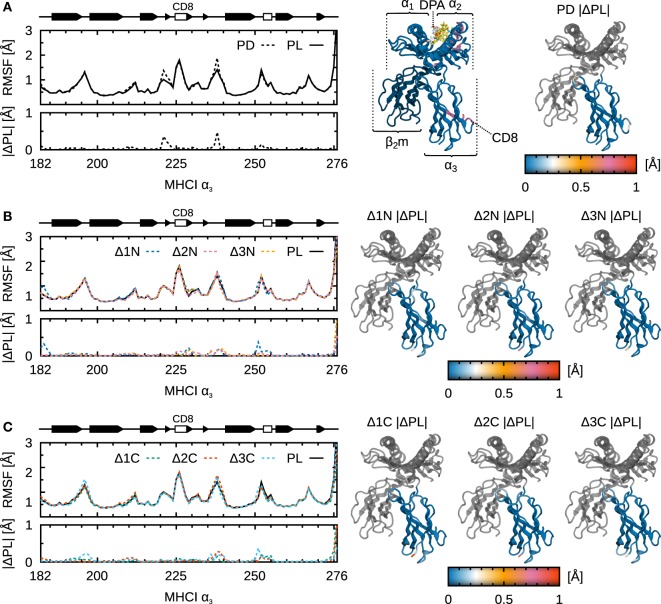
Influence of peptide cargo on B*44:02 α_3_ fluctuations. Cα RMSF along sequence and the absolute difference compared to MHCI^PL^ (|ΔPL|) are shown for each system. ΔRMSF are also mapped onto the structure of α_3_. Peptide not shown for clarity. **(A)** MHCI^PD^. **(B)** MHCI^Δ1N–Δ3N^. **(C)** MHCI^Δ1C–Δ3C^.

To understand which domains of B*44:02 are influenced by peptide truncation and to what extent, we analyzed protein C_α_ RMSF for each system under study. These are shown along B*44:02 sequence in Figure 5 for the α_1_α_2_ binding groove domain, and in Figure 6 for the α_3_ domain. As expected, the binding groove is the region whose fluctuations are most impacted by peptide cargo modification. The α_2−1_ helix segment known to interact with tapasin (7, 14, 15) and the N-terminal portion of α_1_ located in the vicinity of the A-pocket are its most affected regions. Stretch 51–54, in α_1_ N-terminus, was proposed as a hinge that pivots in the transition from peptide-receptive to peptide-loaded MHCI (30, 31) in murine allele H2-L^d^. In previous work (14, 15), we proposed a peptide-editing mechanism according to which peptides and tapasin compete for the α_2−1_ region. The results presented here yield a refined view, in the sense that they suggest there could be two sensing mechanisms at work to distinguish properly loaded MHCI at the A- and F-pockets. The fluctuations in the two affected regions are independent: N-terminal peptide truncation recovers the increased RMSF in α_1_ (Figure 5B), while C-terminal truncation yields increased fluctuations in α_2−1_ (Figure 5C).

Possible long-range effects of cargo modification on α_3_ are important to consider given that this domain contacts tapasin in the PLC and that allosteric effects involving α_3_ have been proposed (24) as a mechanism for peptide sensing by tapasin. However, apart from the Δ1N peptide (see below), we could not observe any significant effect of peptide truncation on α_3_ fluctuations (Figure 6). It is possible, however, that signal transduction events are slow compared to the length of our trajectories (1.0 µs) and that increased sampling could yield a different picture. Another possibility is that subtle conformational changes in α_3_ are not reflected in the magnitude of its fluctuations.

### 3.3 Configurational Entropy As a Function of Peptide Cargo

**Figure 7:**
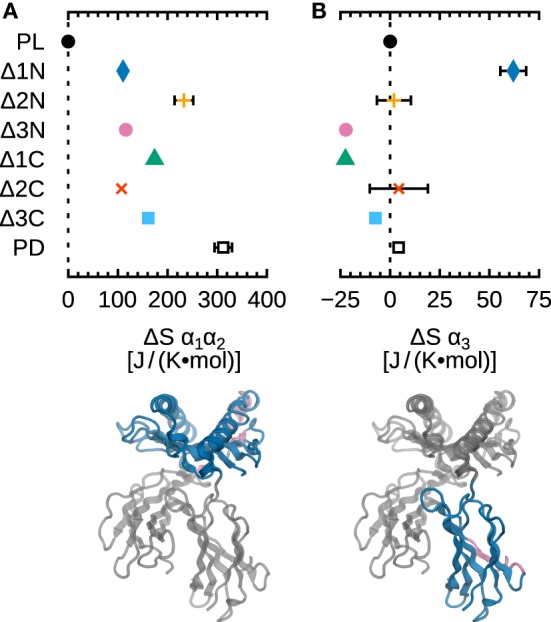
Influence of peptide cargo on B*44:02 configurational entropy. ΔS for each system is the difference to MHCI^PL^ computed for the given domain. **(A)** α_1_α_2_. **(B)** α_3_. Statistical error estimates are the difference between ΔS computed from the full trajectories to the value obtained when discarding a random 10% of the data, and are shown only when larger than 10 J/(K mol).

To complement our analysis of fluctuations, we computed configurational entropies for the α_1_α_2_ and α_3_ domains of B*44:02. Results are shown in Figure 7, where ΔS is the difference between the entropy of each system and the entropy of the reference system MHCI^PL^. The important increase of entropy (100–300 J/[K mol]) observed for α_1_α_2_ (Figure 7A) is consistent with the increased fluctuations of the individual residues, as discussed above. The small statistical uncertainties (less than ±10 J/[K mol] for most systems) indicate convergence, i.e., that 5.0 µs of MD sampling is sufficient to assess B*44:02 α_1_α_2_ dynamics.

Configurational entropy differences in the α_3_ domain (Figure 7B) are also consistent with residue RMSF: ΔS is very small for most systems. A modest but significant increase (63 J/[K mol]) is observed, however, for Δ1N. While this could be an indication of a signal being transmitted between the binding groove and α_3_, it also raises the question of why this difference is not observed for any other N- or C-terminal deletion. One possibility is that, since allostery-related protein dynamics often take place on the microsecond–millisecond timescale, our simulations lack the sampling necessary to describe such slow events. Another possibility is that a certain stiffness of the binding groove is required for signal transduction, and that the increased α_1_α_2_ plasticity caused by deletions larger than a single residue may disrupt this sensing mechanism; however, this would not explain the apparent absence of allosteric signal in the Δ1C system. Finally, a third possibility is that the presence of tapasin, the tapasin–ERp57 conjugate, or other accessory proteins forming the PLC, is necessary to induce conformational changes in MHCI that mediate the allosteric mechanism.

### 3.4 Contributions of the Peptide Side Chains and Backbone

Antigenic peptide specificity is often driven by their N- and C-terminal residues (65), which also determine their stability in the MHCI binding groove. In B*44:02^DPA^, residues E2 and F9 extend their side chains toward the floor of the binding groove, occupying the A- and F-pockets, respectively. Since their truncation leads to partial peptide dissociation in our simulations (see Figure 2), we expect their side chains to be key determinants of peptide stability. To test this hypothesis, we have carried out additional simulations of B*44:02-bound modified DPA peptides. These have had one or two residues replaced by glycines at either the N- or the C-terminus. We denote these systems MHCI^δ1N^, MHCI^δ2N^, and MHCI^δ1C^. As for the other modified peptides, trajectories totaling 5 µs of MD simulations (5 × 1 µs) were acquired for each system.

**Figure 8:**
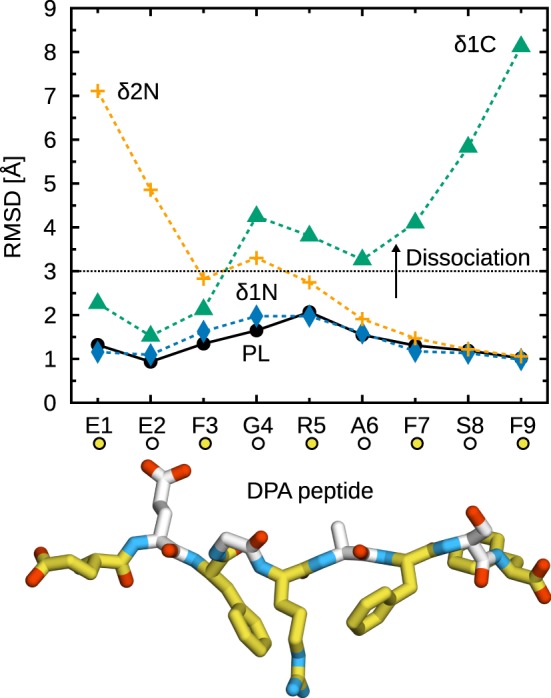
Partial dissociation of peptides with glycine substitutions from B*44:02. C_α_ RMSD from the initial position in the X-ray crystal structure were averaged for each peptide residue over the five independent 1-µs trajectories acquired for each system. The dotted line represents a chosen dissociation threshold at 3 Å.

Partial peptide dissociation was observed for two of the three systems under study (Figure 8). Interestingly, side chain removal at the N-terminus leads to a partial dissociation pattern that is significantly different from the one observed for the corresponding truncated peptides. δ1N does not dissociate at all, showing a stable binding almost identical to that of unmodified DPA. δ2N exhibits partial dissociation, but to a much lesser extent than Δ2N: only E1 and E2 vacate the binding groove. Therefore, at the N-terminus, both the peptide main chain and the E1 and E2 side chains contribute to MHCI binding at the A-pocket. Conversely, at the C-terminus, the δ1C dissociation pattern is similar to that of Δ1C, with residues 4–9 dissociating from the groove. The F9 side chain is thus a critical determinant of peptide binding at the F-pocket.

### 3.5 Tapasin Widens the Binding Groove of MHCI and Weakens Peptide Binding Forces

**Figure 9:**
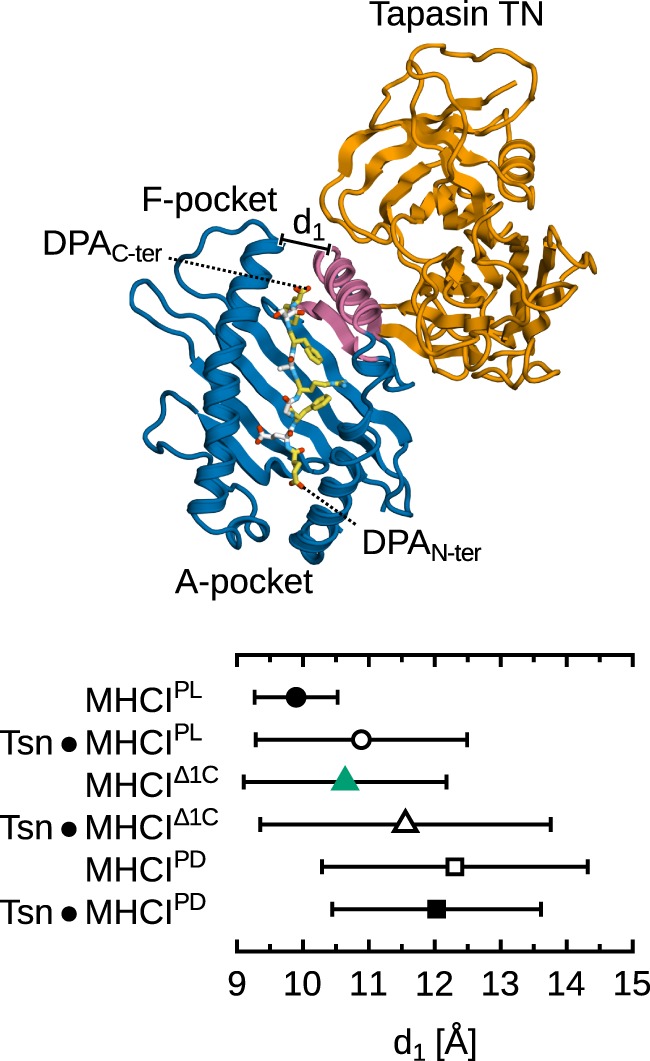
**Effect of tapasin on MHCI-binding groove width near the F-pocket**. Distance d_1_ is measured between the C_α_ atoms of Y85 and T138 as in Figure 4. Distances were averaged over all simulations for each system; standard deviation is shown as bars. Standard errors of the mean, obtained from a block averaging procedure (64), are below 0.5 Å in all cases. The tapasin–MHCI controls for the PL and PD forms are taken from previous simulations (15). Tapasin TC and MHCI α_3_ not shown for clarity.

We performed 5 additional, independent, 1-µs MD simulations of MHCI^Δ1C^ in complex with tapasin, using our previously determined structure of tapasin–MHCI (14, 15) and truncating the full-length DPA peptide present in that structure. This allows us to compare the dynamics of B*44:02 loaded with a low-affinity peptide in the free and tapasin-complexed forms. Results (Figure 9) show that the binding groove width near the F-pocket (d_1_, as shown previously in Figure 4), is wider by about 1.0 Å in the presence of tapasin. This is consistent with the “tug-of-war” mechanism we previously described (15), according to which tapasin selects peptides depending on their C-terminus by pulling MHCI β_7_ underneath α_2−1_ to open the groove and thus accelerate peptide release.

**Figure 10:**
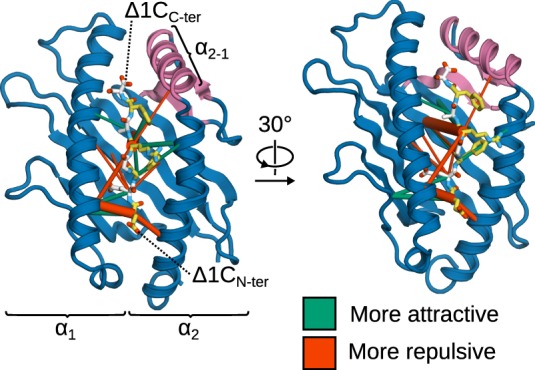
**Effects of tapasin on the intermolecular forces in the binding groove of MHCI^Δ1C^**. Differences (ΔF) in the pairwise residue-residue forces between tapasin-complexed and -free MHCI are shown as cylinders scaled according to force difference magnitude, and show the effects of transitioning MHCI^Δ1C^ from the tapasin-free form to the tapasin-complexed one. Force differences range from 30 to 150 pN; smaller differences are statistically insignificant, and therefore not shown. MHCI α_3_ not shown for clarity.

Next, to obtain a more detailed and spatially resolved picture, we used force distribution analysis (FDA) to compute the residue-residue pairwise forces between B*44:02 and the Δ1C peptide in tapasin-complexed and -free MHCI (Figure 10). Overall, forces between MHCI and the peptide are on average less attractive (by 150 pN) in the tapasin–MHCI complex, showing that the opening of the groove induced by tapasin decreases the affinity of MHCI for its low-affinity cargo. Interestingly, considering individual pairwise forces at the residue level reveals that tapasin affects the peptide not just in the vicinity of the α_2−1_ region (C-terminus), but along its whole sequence. Indeed, the largest force difference is observed between the peptide at position R5 and B*44:02 R97 at the bottom of the binding groove; in the tapasin–MHCI complex, this force is more repulsive (by 148 pN) than observed for B*44:02 in the free form. A less attractive binding force is also observed at the peptide N-terminus (E1). Thus, tapasin-induced widening of the binding groove near the F-pocket (see Figure 9) has long-ranging effects that destabilize peptide binding, increasing the latter’s off-rate. Full peptide release, however, was not observed in the present simulations. Much longer timescales would likely be required to investigate the tapasin-catalyzed unbinding event itself.

Binding groove widening has been suggested before (9) in a biochemical study of tapasin–MHCI. In their mechanism of complex assembly/disassembly, the authors proposed two possible conformational transitions taking place in the MHCI binding groove. The first is an equilibrium between “closed” and “open” states, which exist both in presence and absence of tapasin and could correspond to a tightly packed and loose binding groove, respectively (9, 24, 66). The second is a switch between a canonical and a “tapasin-disrupted” binding groove conformation, which could correspond to the widening we observe in our simulations. Partial peptide dissociation of the Δ1C peptide, in turn, would prevent the transition from the “open” to the “closed” state. Interestingly, the same study (9) also found that mutating certain high-affinity peptides at their N-terminus caused them to exhibit higher tapasin sensitivity. This indicates that tapasin impacts MHCI across the full length of the peptide, which is consistent with our above FDA results that reveal destabilizing effects at E1 and R5.

## 4 Discussion

Using comparative MD simulations of B*44:02 loaded with a variety of truncated DPA peptides, we have shown that positions 2 and 9 are crucial for peptide stability in the binding groove; the corresponding Δ2N and Δ1C are the smallest deletions leading to partial dissociation of the peptide central residues on the microsecond timescale. Even in the full-length antigen, however, central peptide residues enjoy substantial flexibility, potentially facilitating recognition by the TCR CDR3 loop. Transient rebinding to the groove indicates that at least some MHCI molecules exist in an equilibrium of two populations: one with a tightly bound peptide and the other with a partially dissociated peptide. These results are consistent with NMR experiments on allele B*35:01 in complex with different variant peptides (63), and could indicate the first step leading to the internalization and recycling of MHCI. Furthermore, partial dissociation from MHCI at the peptide N-terminus could explain how ERAAP can trim MHCI-bound peptides to their correct final length (19). Finally, partial binding at the N-terminus is likely the first step in dipeptide-catalyzed peptide exchange (36), where the MHCI F-pocket is occupied by short dipeptides that are rapidly replaced upon the addition of high-affinity peptides.

Peptide truncation impacts B*44:02 dynamics in a different manner than complete peptide removal does, especially for N-terminal deletions. This suggests that MHCI^PD^ is a sub-optimal surrogate for MHCI bound to low-affinity peptides, something that needs to be considered for simulation studies of peptide editing, especially N-terminal editing by ERAAP. MHCI^ΔC^ systems, on the other hand, are more similar to the PD form as far as binding groove dynamics in the vicinity of the F-pocket are concerned. Fluctuation and entropy analyses confirm the importance of the α_2−1_ helix segment bordering the F-pocket of the binding groove. Increased flexibility (as observed for the ΔC systems) is necessary for C-terminal peptide editing by the tapasin TN domain as described previously (15): tapasin and the antigen compete for α_2−1_ to open and close the groove, respectively, leading to accelerated peptide release until the binding of a high-affinity antigen.

MHCI B*44:05, which differs from B*44:02 by only one residue (Y116 instead of D), can efficiently load antigens without tapasin. Since Y116 is located on the floor on the binding groove (β_6_) underneath the α_2−1_ helix segment, it is reasonable to expect it to influence the dynamics of that region. Indeed, comparative MD simulation studies (27) of these two alleles have found that B*44:05 exhibits less fluctuations in the vicinity of the F-pocket. By enforcing partial dissociation of the N- and C-terminal residues through umbrella sampling, the authors found that the energy barrier required for peptide dissociation at the C-terminus is higher in B*44:05 (tapasin-independent) than in B*44:02 (tapasin-dependent). A similar conclusion was drawn for alleles B*27:05 and B*27:09 (32), that also differ by a single amino acid in the F-pocket: tapasin-dependent B*27:05 exhibits higher conformational flexibility in the vicinity of the α_2−1_ region. Therefore, dependence on tapasin could stem, at least for these two alleles, from a requirement for its chaperone activity, which confines MHCI to a peptide-receptive conformation. The widening of the binding groove, conversely, relates apparently only to the catalysis of peptide dissociation. Future work involving the simulation of tapasin-independent allele B*44:05 with modified or truncated peptides is required to determine if partial peptide dissociation at the C-terminus follows a similar pattern as in B*44:02.

We found no strong indication of allosteric effects of peptide truncation on the CD8 loop contacting tapasin. We did, however, observe a possible modulation of α_3_ dynamics in the Δ1N peptide system. More extensive sampling might be required to elucidate α_3_ dynamics and reconcile MD simulations with the computational systems models of tapasin function that were recently proposed (24). If C-terminal peptide editing proceeds by a dual mechanism, the next pertinent question would be what is the purpose of each mechanism. One possible hypothesis is that the TN–α_1_α_2_ interface provides the mechanical force to open the groove, and therefore accelerates peptide release; the TC–α_3_ one is a signal for tapasin to recognize MHCI loaded with low-affinity cargo and disengage once a high-affinity antigen is bound. Extended MD simulations of the tapasin–MHCI complex, comparing the effects of low- and high-affinity peptides, could be used to test this hypothesis at the atomic level.

## Author Contributions

OF and LS designed the study. OF, SW, and LS analyzed and interpreted the results and revised the manuscript. OF performed the simulations and drafted the manuscript.

## Conflict of Interest Statement

The authors declare that the research was conducted in the absence of any commercial or financial relationships that could be construed as a potential conflict of interest.
